# Solitary Sarcoid Granuloma of the Iris Mimicking Tuberculosis: A Case Report

**DOI:** 10.1155/2014/656042

**Published:** 2014-03-10

**Authors:** Robert Rejdak, Pavel Pogorelov, Christian Y. Mardin, Małgorzata Szkaradek, Anselm G. M. Juenemann

**Affiliations:** ^1^Department of General Ophthalmology, Medical University of Lublin, 20-079 Lublin, Poland; ^2^Medical Research Center of the Polish Academy of Sciences, 02-106 Warsaw, Poland; ^3^Department of Ophthalmology, University of Erlangen-Nürnberg, 91054 Erlangen, Germany; ^4^Department of Ophthalmology, F. Chopin Specialist District Hospital in Rzeszów, 35-055 Rzeszów, Poland

## Abstract

*Introduction*. We report a case of a male patient presented with sarcoid lesions of the iris and conjunctiva, mimicking tuberculosis due to epithelioid cell granulomas with small central necrosis in conjunctival biopsy. *Patient*. A 25-year-old man was referred to our department for further management of an “iris tumor with iridocyclitis” in his right eye. Initial examination showed an isolated vascular tumor of the iris and ciliary body with anterior uveitis and mutton-fat keratic precipitates, suggesting the diagnosis of a granulomatous disease. Conjunctival biopsy revealed granulomatous epithelioid cell inflammation with small central necrosis without acid-fast bacilli. Extensive systemic examination, including bronchoscopy and transbronchial biopsy, provided the diagnosis of sarcoidosis stage 2 with pulmonary involvement, thus ruling out tuberculosis. Systemic and local steroid therapy was initiated, leading to complete recovery of our patient with complete disappearance of the iris lesion and improvement of the pulmonary function. *Conclusion*. Although noncaseating epithelioid cell granulomas are typical for sarcoidosis, small central necrosis can be found in some granulomas, leading to presumption of tuberculosis. Extensive systemic checkup in cooperation with other specialists is essential to confirm the correct diagnosis and to initiate the appropriate therapy.

## 1. Case Presentation

A 25-year-old Caucasian male patient presented with a three-week history of a red right eye. He complained of blurred vision on the affected eye without pain or other sensations. He had no fever, fatigue, and denied weight loss. His past medical and family history were unremarkable.

**Figure 1:**
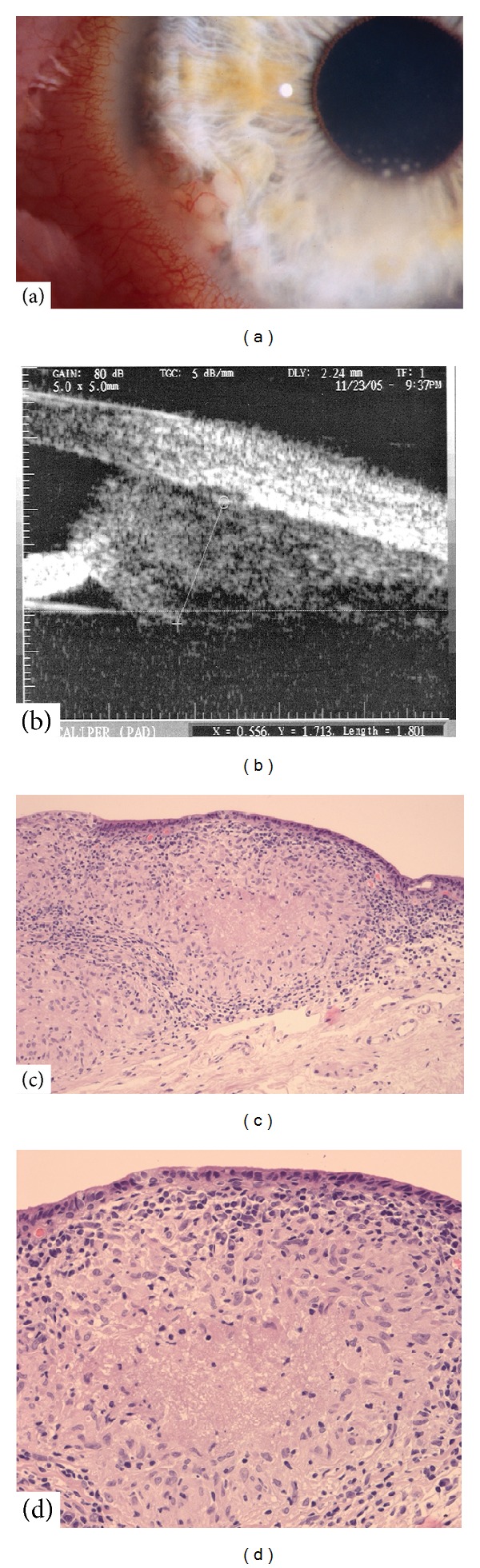
(a) Slit-lamp examination of prominent vascularized lesion of the iris and ciliary body with keratic precipitates. (b) Ultrasound biomicroscopy (UBM) image of the iris and ciliary body lesion. (c and d) Conjunctival biopsy specimen shows epithelioid cell granulomaswith central necrosis (periodic acid-Schiff staining).

On examination, the patient's visual acuity was 14/20 OD and 20/20 OS. A prominent vascularized iris lesion located at 7 o'clock was present in the right eye in addition to mutton-fat keratic precipitates, predominantly in Arlt's triangle, anterior chamber cells, and mixed conjunctival and ciliary injection (Figure 1(a)). Neither fundoscopy of the right eye nor examination of the left eye disclosed any pathological findings.

An ultrasound biomicroscopy (UBM) of the right eye revealed a prominent iris process with extension into ciliary body which measured 4.7 × 4.0 × 1.8 mm with middle internal reflectivity (Figure 1(b)).

Laboratory findings were unremarkable including serum ACE level and tuberculin testing. Chest computed tomography (CT) revealed bilateral hilar lymphadenopathy and reticulonodular parenchymal involvement.

To confirm the suggested diagnosis of sarcoidosis, conjunctival biopsy was performed. Histopathological work-up showed granulomatous epithelioid cell inflammation with insular central necrosis without acid-fast bacilli (Figures 1(c) and 1(d)). The diagnosis of tuberculosis was suspected and further examinations were initiated.

Extensive systemic checkup, including bronchoscopy, bronchoalveolar lavage (BAL) with CD4/CD8 ratio evaluation, and transbronchial biopsy, revealed no evidence of acid-fast organisms. Transbronchial biopsy showed noncaseating granulomatous inflammation, typical for sarcoidosis. PCR showed no mycobacterial DNA in the biopsy specimen.

Systemic and topical steroid therapy was initiated with oral prednisolone 100 mg daily and prednisolone acetate 1% eye drops hourly. Under this treatment our patient recovered completely. Eight weeks after the first presentation, no signs of intraocular inflammation were detectable, the iris tumor resolved completely, and the patient was free of any complaints.

## 2. Discussion

Sarcoidosis is a chronic multisystem granulomatous disorder of unknown origin [1, 2]. Sarcoidosis occurs worldwide but is predominant in certain ethnic and racial groups (e.g., Scandinavians and US blacks) and usually develops before the age of 50 years with the incidence peaking at 20 to 39 years [3]. The highest annual incidence of sarcoidosis has been observed in northern European countries (5 to 40 cases per 100,000 people) [4]. Sarcoidosis is characterized by the formation of noncaseating granulomas in affected organs, predominantly in the lungs and thoracic lymph nodes (more than 90% of patients) and skin (25% of patients), and might have either self-limited (two thirds of patients) or chronic course (up to one third of patients) [2, 5]. Less than 5% of patients die from sarcoidosis [5]. Ocular involvement occurs in 25–60% of patients with systemic sarcoidosis. The most common ocular manifestations are uveitis (30–70%) and conjunctival nodules (40%). Classic sarcoid associated anterior uveitis may present either as acute iridocyclitis, which is mostly seen in Löfgren's syndrome, or as a chronic granulomatous uveitis with mutton-fat keratic precipitates [2]. In the chronic type of the disease, the granulomatous nodules of the iris and in the anterior chamber angle are sometimes seen (the so-called Koeppe and Busacca nodules). In rare cases, iris sarcoid granulomas can simulate other prominent iris lesions such as an amelanotic melanoma of the iris or a carcinoma metastasis [6, 7] such as in our case.

In the absence of the known causative agent, the diagnosis of sarcoidosis remains mainly a diagnosis of exclusion. Chest computed tomography has been reported to be more sensitive than radiography and can show hilar adenopathy in combination with interstitial lung disorder. Measurements of serum angiotensin converting enzyme (ACE) level reflect the disease activity but are not specific enough for diagnostic properties; normal serum ACE levels do not exclude the diagnosis of sarcoidosis, especially not in case of isolated ocular manifestation.

Clinical features together with the presence of noncaseating epithelioid cell granulomas on tissue biopsy are usually pathognomonic for the diagnosis of sarcoidosis. Tissue biopsy might be obtained from lung, skin, lymph node, or other affected tissues such as conjunctiva. Conjunctival biopsy has been shown to be positive in 66.7% in patients with and in 31.4% without conjunctival follicles [8].

Although noncaseating granulomas are not pathognomonic, tissue examination is essential to differentiate sarcoidosis from infections or malignancies [1, 9]. In our case, small central necrosis within epithelioid cell granulomas was present, mimicking caseating tuberculous granulomas. A similar case of patient with scrotal sarcoid granulomas with central necrosis, leading to presumption of tuberculosis, has also been reported [10].

The ideal therapy for sarcoidosis is not well defined; therapeutic decisions are dictated by the localisation and severity of the disease. Nevertheless, oral corticosteroids are the mainstay of treatment. For refractory course and in some cases of ocular involvement, methotrexate was reported to be effective.
